# NAP: The Network Analysis Profiler, a web tool for easier topological analysis and comparison of medium-scale biological networks

**DOI:** 10.1186/s13104-017-2607-8

**Published:** 2017-07-14

**Authors:** Theodosios Theodosiou, Georgios Efstathiou, Nikolas Papanikolaou, Nikos C. Kyrpides, Pantelis G. Bagos, Ioannis Iliopoulos, Georgios A. Pavlopoulos

**Affiliations:** 1 Bioinformatics & Computational Biology Laboratory, Division of Basic Sciences, University of Crete Medical School, 70013 Heraklion, Crete, Greece; 2 Joint Genome Institute, Lawrence Berkeley Lab, United States Department of Energy, 2800 Mitchell Drive, Walnut Creek, CA 94598 USA; 3 Department of Computer Science and Biomedical Informatics, University of Thessaly, Papasiopoulou 2-4, Galaneika, 35100 Lamia, Greece

**Keywords:** Network biology, Network topology, Node and edge ranking, Centralities, Network comparison

## Abstract

**Objective:**

Nowadays, due to the technological advances of high-throughput techniques, Systems Biology has seen a tremendous growth of data generation. With network analysis, looking at biological systems at a higher level in order to better understand a system, its topology and the relationships between its components is of a great importance. Gene expression, signal transduction, protein/chemical interactions, biomedical literature co-occurrences, are few of the examples captured in biological network representations where nodes represent certain bioentities and edges represent the connections between them. Today, many tools for network visualization and analysis are available. Nevertheless, most of them are standalone applications that often (i) burden users with computing and calculation time depending on the network’s size and (ii) focus on handling, editing and exploring a network interactively. While such functionality is of great importance, limited efforts have been made towards the comparison of the topological analysis of multiple networks.

**Results:**

Network Analysis Provider (NAP) is a comprehensive web tool to automate network profiling and intra/inter-network topology comparison. It is designed to bridge the gap between network analysis, statistics, graph theory and partially visualization in a user-friendly way. It is freely available and aims to become a very appealing tool for the broader community. It hosts a great plethora of topological analysis methods such as node and edge rankings. Few of its powerful characteristics are: its ability to enable easy profile comparisons across multiple networks, find their intersection and provide users with simplified, high quality plots of any of the offered topological characteristics against any other within the same network. It is written in R and Shiny, it is based on the igraph library and it is able to handle medium-scale weighted/unweighted, directed/undirected and bipartite graphs. NAP is available at http://bioinformatics.med.uoc.gr/NAP.

## Introduction

Metabolic reactions, signal transduction, gene expression, gene regulation, protein interactions and other biological concepts are often captured in network representations showing individual bioentities as nodes and their interconnections as edges. Each network is characterized by a different topology. In small-world networks for example, any node in the graph can be reached from any other node in a small number of steps. In scale-free networks, highly connected nodes can be identified as hubs. Networks with densely connected neighborhoods have high clustering coefficient and tend to form clusters. In social networks, the robustness is sensitive upon edges with high betweenness centrality, necessary to bridge distant communities. Protein–protein interaction networks (PPIs) are captured as undirected connected graphs following a scale-free topology with hierarchical modularity [1, 2].

While existing visualizations often comply with topological network analysis [3–6], only few of them purely focus on topological analysis, comparison and edge/node ranking. Cytoscape’s [7] Network Analyzer [8] as well as Gephi [9], offer similar functionality but do not support direct comparison between topological features of multiple networks. ZoomOut [10] and Network Analysis Toolkit (NEAT) [11] on the other hand are mostly focused on graph clustering. Stanford Network Analysis Platform (SNAP) [12] and igraph [13] offer a wide spectrum of functions and modules related to topological analysis but are offered as command line libraries, thus making them less accessible to non-experts.

To overcome these barriers, we offer NAP, a modest web application, dedicated to make network topological analysis and inter/intra-network topological comparison simpler and more appealing to the broader community.

## Main text

### The GUI

NAP comes with a self-explanatory web interface, organized in several tabs.

#### Upload file tab

**Fig. 1:**
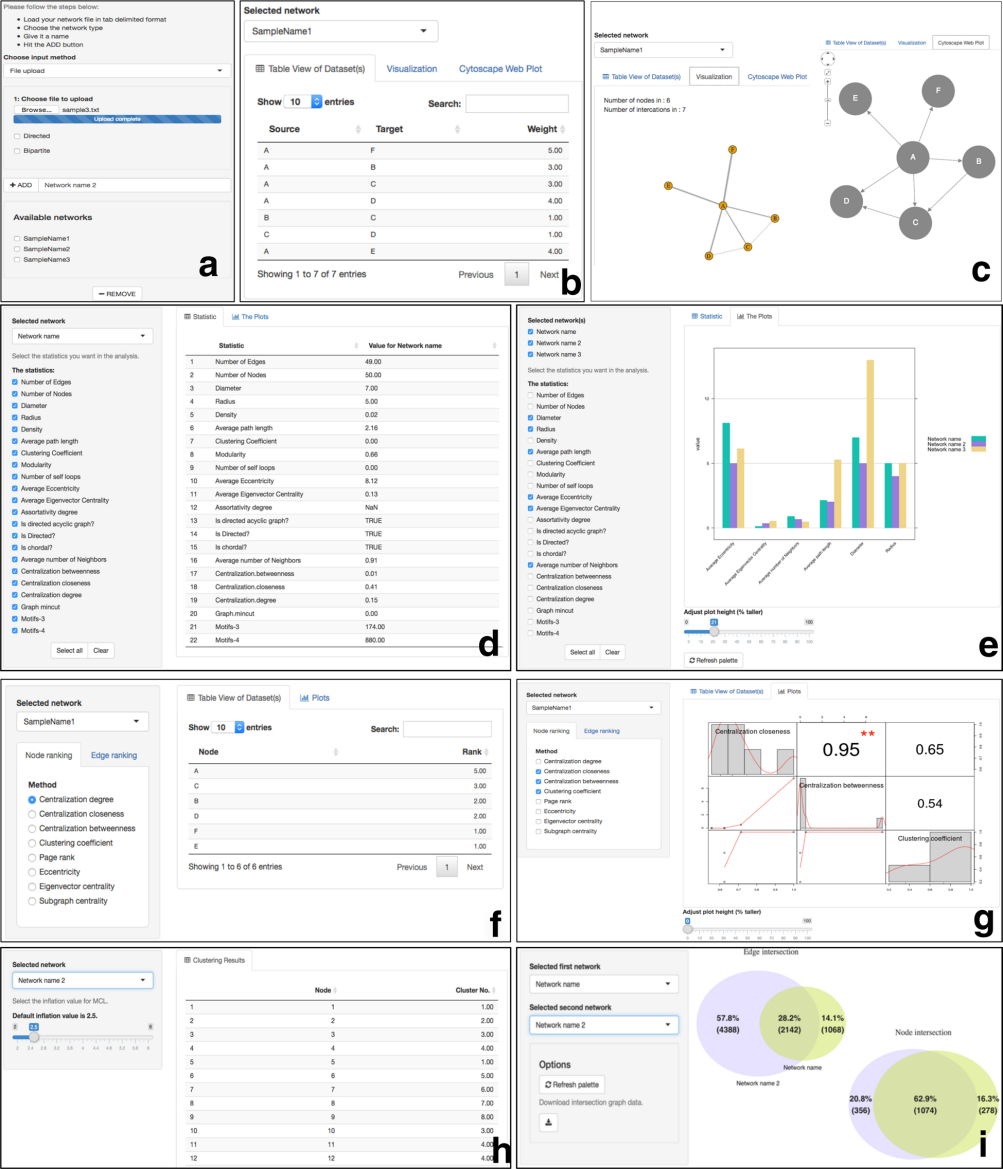
NAP’s web interface. **a** Users can upload several networks in the form of a list (pairwise connections) and subsequently name them. Users can also generate graphs of various sizes (50, 100, 1000, 10,000) based on the Barabási–Albert, Erdos–Renyi or Watts–Strogatz small-world model. Additionally, users can generate bipartite graphs of various sizes. **b** Network contents in the form of searchable and sortable tables. **c**-*left* Static network visualization. **c**-*right* Interactive Cytoscape.js network visualization. **d** Selection of topological features and their values. **e** Inter-network comparisons of topological features. **f** Node/edge ranking in the view of searchable tables. **g** Intra-network topological feature comparison in the form of a matrix. **h** Implementation of MCL clustering algorithm. **i** Intersection of any two chosen networks

It is dedicated to file uploading and network naming (Fig. 1a). Once one or more networks have been uploaded, three sub-tabs will appear. In the first sub-tab, users can see the network as a binary list in the form of searchable tables (Fig. 1b), in the second sub-tab a static visualization of the network and in the third sub-tab an interactive network visualization (Fig. 1c).

#### Topology tab

The second tab is dedicated to network topological analysis. Once one or more networks are loaded, users can interactively choose between several topological features. While, here, users can explore one network at a time, in a second sub-tab users can automatically generate an inter-network topological analysis plot in order to directly compare one or more networks. Examples of these cases can be depicted in Fig. 1d, e.

#### Ranking tab

This part is dedicated to node and edge ranking. Users can interactively choose between several node and edge topological features and sort the relevant nodes/edges accordingly. Moreover, users can plot the distribution of any topological feature of a network against any other and visualize it in a matrix-like plot. Examples are presented in Fig. 1f, g.

#### Clustering tab

This tab is dedicated to network clustering. While NAP is not intended to be a network clustering application, MCL Markov Clustering is incorporated [14]. This way, user can cluster medium-sized networks (Fig. 1h).

#### Intersection

This tab is dedicated in calculating the intersection between any pair of selected networks. Results are shown as Venn diagrams and can an export function to download the intersecting network is offered (Fig. 1i).

### Input file

NAP supports loading of multiple weighted/unweighted, directed/undirected and bipartite graphs. Each network can be loaded as a two-column binary list of connections as a tab delimited text file. After uploading, users must manually give a name and define the type of each network. In addition, random networks of various sizes (100, 1000, 10,000 nodes) and types (Barabási–Albert, Erdos–Renyi, Watts–Strogatz small-world and bipartite graphs) can be automatically generated and used as examples. Notably, NAP currently accepts networks of up to 50,000 edges.

For this article, we used two yeast protein–protein interaction (PPI) networks: Gavin 2006 [15] and Gavin 2002 [16], the first consisting of 6531 edges and 1430 vertices and the second consisting of 3210 edges and 1352 vertices. For the first dataset, large-scale tandem affinity purification and mass spectrometry were used to characterize multiprotein complexes in *Saccharomyces cerevisiae* whereas the second dataset shows the first genome-wide screen of complexes in *Yeast*.

### Basic visualization

Nodes and edges can be presented as dynamic, easy to filter, excel-like tables, as well as static and dynamic 2D network visualizations. Tables are sortable by name and searchable using simple substring matching.

#### Static visualization

While NAP is not designed to be a visualization tool, its 2D static network visualization comes with a plethora of traditional layout algorithms (*Random, Circle, Sphere, Fruchterman*–*Reingold, Reingold*–*Tilford, Kamada*–*Kawai, Grid, Lgl and SVD*). After a completed layout, nodes and their coordinates, along with their connections can be exported as simple text files and imported to other, more advanced visualization tools [3–6].

#### Dynamic visualization

NAP utilizes CytoscapeWeb/Cytoscape.js [17, 18]. to additionally provide a dynamic network visualization. Users can interactively zoom in/out, relocate the nodes and select them and choose between various edge/node colors and shapes and among very standard graph layouts.

We chose to provide both static and dynamic visualization at a basic level so that the user can get an at-a-glance view of the loaded network. Notably, NAP’s visualization cannot scale very well due to browser limitations but is fair for middle-sized networks. For higher quality visualization, graph editing, manipulation and interactive network exploration, users are encouraged to use other available tools such as Cytoscape and Gephi. The input file format for NAP, Cytoscape and Gephi is the same (2 column tab delimited file).

### Topological features

NAP is able to calculate several topological features for a selected network taken from the igraph library. While in igraph’s manual pages one can find more detailed information about the calculations, most formulas and definitions are also explained in [19]. Table 1 summarizes a simplified explanation of NAP’s aforementioned metrics.

**Table 1:** NAP’s supported topological features and their explanation

Topological feature	Simplified explanation
Number of edges	Shows the number of edges in the network. Moderate network of several thousand connections are very acceptable
Number of nodes	Shows the number of nodes in the network. There is no limitation on the number of nodes
Diameter	Shows the length of the longest geodesic. The diameter is calculated by using a breadth-first search like method. The graph-theoretic or geodesic distance between two points is defined as the length of the shortest path between them
Radius	The eccentricity of a vertex is its shortest path distance from the farthest other node in the graph. The smallest eccentricity in a graph is called its radius. The eccentricity of a vertex is calculated by measuring the shortest distance from (or to) the vertex, to (or from) all vertices in the graph, and taking the maximum
Density	The density of a graph is the ratio of the number of edges and the number of possible edges
Number of edges	Shows the number of edges in the network. If the has more than 10,000 edges it will take into account the first 10,000
Average path length	The average number of steps needed to go from a node to any other
Clustering coefficient	A metric to show if the network has the tendency to form clusters
Modularity	This function calculates how modular is a given division of a graph into subgraphs
Number of self-loops	How many nodes are connected to themselves
Average eccentricity	The eccentricity of a vertex is its shortest path distance from the farthest other node in the graph
Average eigenvector centrality	It is a measure of the influence of a node in a network
Assortativity degree	The assortativity coefficient is positive is similar vertices (based on some external property) tend to connect to each, and negative otherwise
Is directed acyclic graph	It returns True (1) or False (0)
Is directed	It returns True (1) or False (0) depending whether the edges are directed or not
Is bipartite	It returns True (1) or False (0) depending whether the graph is bipartite or not
Is chordal	It returns True (1) or False (0). A graph is chordal (or triangulated) if each of its cycles of four or more nodes has a chord, which is an edge joining two nodes that are not adjacent in the cycle. An equivalent definition is that any chordless cycles have at most three nodes
Average number of neighbors	How many neighbors each node of the network has on average
Centralization betweenness	It is an indicator of a node’s centrality in a network. It is equal to the number of shortest paths from all vertices to all others that pass through that node. Betweenness centrality quantifies the number of times a node acts as a bridge along the shortest path between two other nodes
Centralization closeness	It measures the speed with which randomly walking messages reach a vertex from elsewhere in the graph
Centralization degree	It is defined as the number of links incident upon a node
Graph mincut	It calculates the minimum st-cut between two vertices in a graph The minimum st-cut between source and target is the minimum total weight of edges needed to remove to eliminate all paths from source to target
Motifs-3	Use of igraph to searches a graph for motifs of size 3
Motifs-4	Use of igraph to searches a graph for motifs of size 4

### Inter-network topological feature comparison

Selected topological features of a single network can be visualized as a multi-column bar chart. This way, a user can for example, see the average closeness centrality, the average clustering coefficient and the average shortest path length of the whole graph as numerical values or as a bar chart. Notably, the chart is dynamic and gets automatically updated upon a selection set of features. When users want to directly compare one or more networks, a combined bar chart with adjusted colors indicating the selected networks, can capture the average topological features of all selected networks next to each other.

**Fig. 2:**
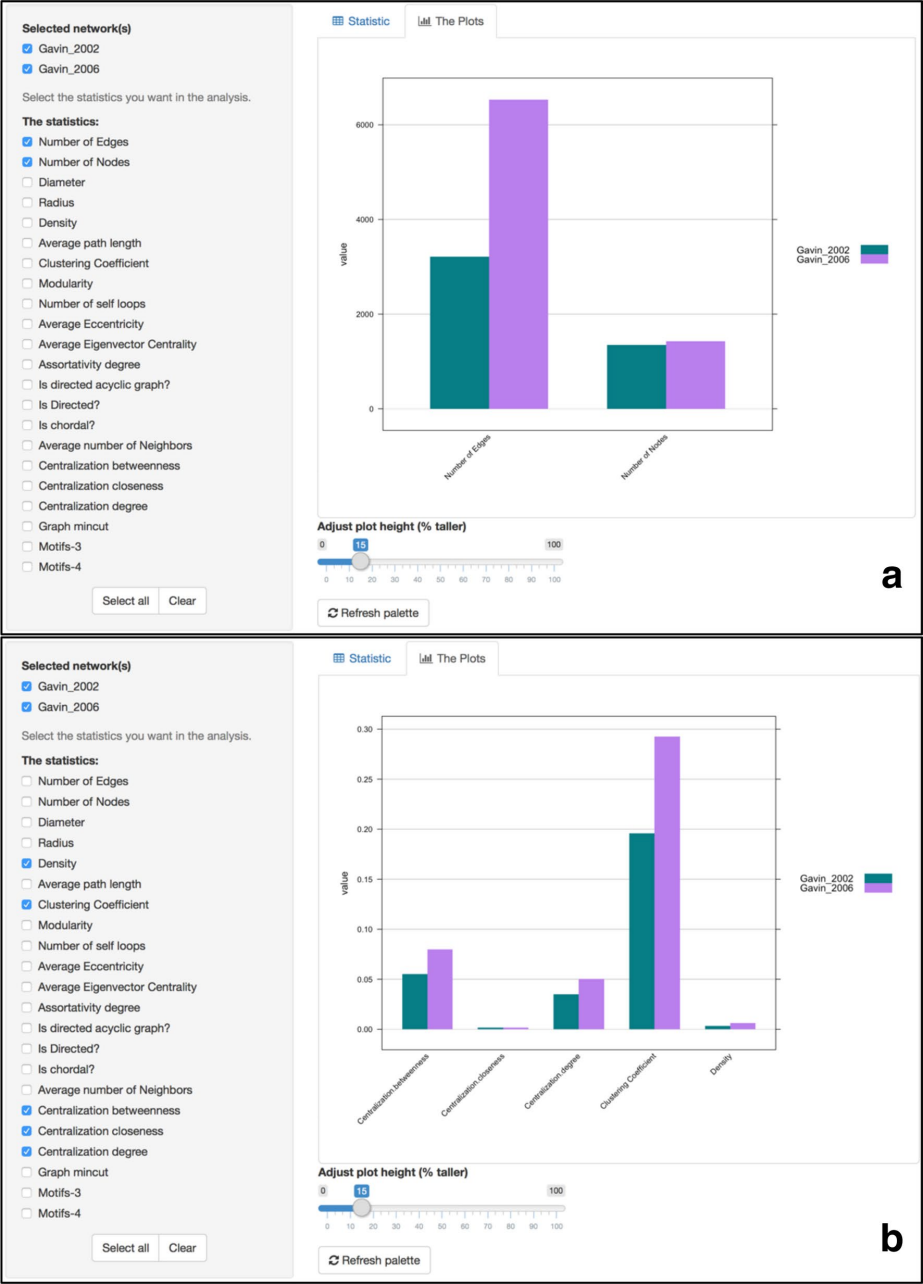
Direct comparison of the topological features of two yeast protein–protein interaction datasets. **a** Gavin 2002 dataset [16] consists of 3210 edges and 1352 vertices, whereas Gavin 2006 [15] consists of 6531 edges and 1430 vertices. **b** Comparison of the networks’ clustering coefficient, density, closeness, betweenness and degree

For example, a straight comparison of the aforementioned yeast protein–protein interaction datasets is presented in Fig. 2. While both networks significantly vary in the number of edges, as shown in Fig. 2a, and despite the fact that they have similar density, they have significantly different clustering coefficient as shown in Fig. 2b. This way Gavin 2006 dataset tends to form tighter clusters compared to Gavin 2002.

### Intra-network topological feature comparison

**Fig. 3:**
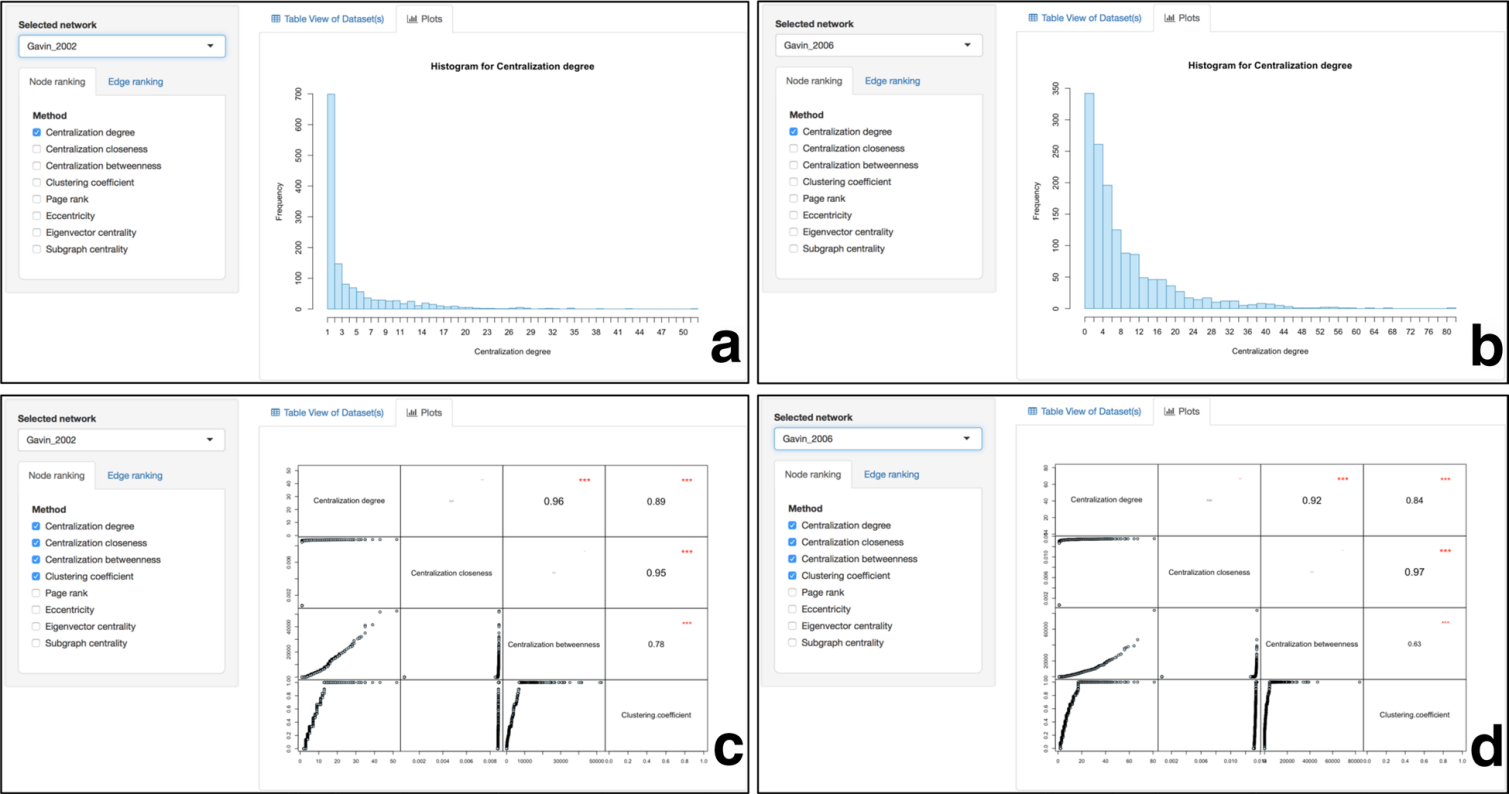
Intra-network comparison of selected topological features within the Gavin 2002 yeast PPI dataset [16]. **a** The degree distribution for Gavin 2002 dataset. **b** The degree distribution for Gavin 2006 dataset. **c** An all-against-all distribution matrix comparing the degree, the closeness, the betweenness and the clustering coefficient for Gavin 2002 PPI network. **d** An all-against-all distribution matrix comparing the degree, the closeness, the betweenness and the clustering coefficient for Gavin 2006 PPI network

Users can select one network at a time and see the distribution of each topological metric. Figure 3a, b for example show the degree distribution for Gavin’s 200 and 2002 PPI network respectively.

In addition, users have the ability to generate a distribution plot showing any topological feature against any other within a selected network. A high-resolution 2D scatterplot is generated on the fly, displaying the distribution of a chosen topological parameter in a histogram-like view. Should the user desire to explore more than one topological parameter at a time, NAP gives the user the opportunity to generate on-the-fly advanced plots by pairwise comparing any topological feature of a network against any other feature within the same network. This matrix-like plot showing pairwise correlations of any combination between the selected topological features is not limited to the number of features to be plotted. The upper triangular part of the plot shows the numerical correlation between any pair of topological features whereas the lower-triangular part of the matrix the scatterplot of one feature against another. The diagonal shows the topological feature which corresponds to that column and row. Like before, two all-against-all plots comparing the degree, the clustering coefficient, the closeness and the betweenness centrality of Gavin 2002 and 2006 PPI datasets are shown in Fig. 3c, d respectively.

Notably, figures can be downloaded as jpeg from the browser while scatter plot coordinates can now be downloaded as CSV files and visualized by external applications like Excel or STATA.

### Node/edge ranking

**Fig. 4:**
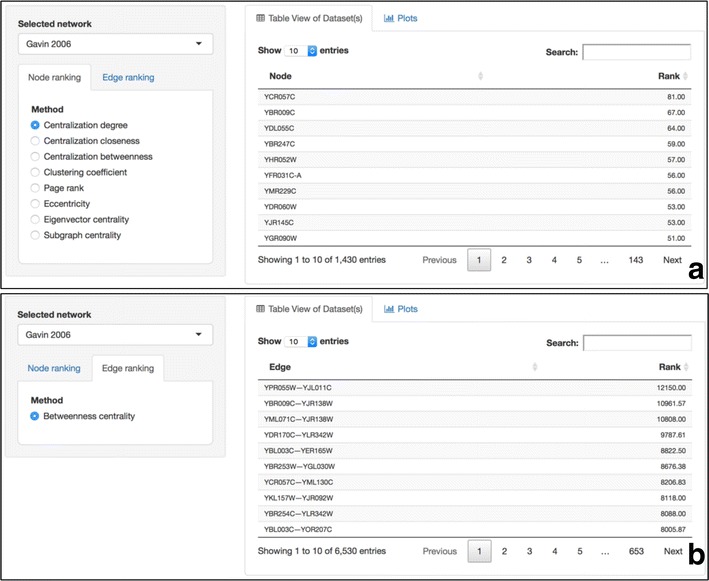
Node and edge ranking. **a** Proteins of the Gavin 2006 PPI datasets are sorted according to their degree. PWP2 (YCR057C) protein has many neighbors and might behave as hub. **b** Interactions of the Gavin 2006 PPI datasets are sorted according to their betweenness centrality. Edge between SEC8 (YPR055W) and RPC17 (YJL011C) behaves as a bridge between communities

Nodes and edges of a selected network (accessible as a drop-down menu) can be sorted according to a preferred topological feature and using dynamic easy-to-filter excel-like tables. Nodes and edges can be sorted in both descending and ascending order. Figure 4a for example shows the proteins of Gavin 2006 PPI network sorted in descending order according to their degree. It is obvious that PWP2 (YCR057C) protein, a conserved 90S pre-ribosomal component essential for proper endonucleolytic cleavage of the 35 S rRNA precursor at A0, A1, and A2 sites is the protein with most connections. Similarly, Fig. 4b shows that the connection between SEC8 (YPR055W) and RPC17 (YJL011C) has the highest betweenness centrality, thus making a very important connection as it acts as a bridge connecting different neighborhoods.

### Clustering

While NAP is not a clustering visualization tool, MCL Markov clustering algorithm has been implemented (Fig. 1h). Users can select a network and adjust the inflation value of MCL. A two-column searchable matrix will be generated showing the node name and the cluster each node belongs two. This way, users can easily find whether two nodes belong in the same cluster or not. This feature is recommended for small and medium-size networks and must be avoided for larger networks. For a deeper clustering analysis, users are encouraged to users command line tools or try the ClusterMaker Cytoscape plugin [20].

### Intersection

**Fig. 5:**
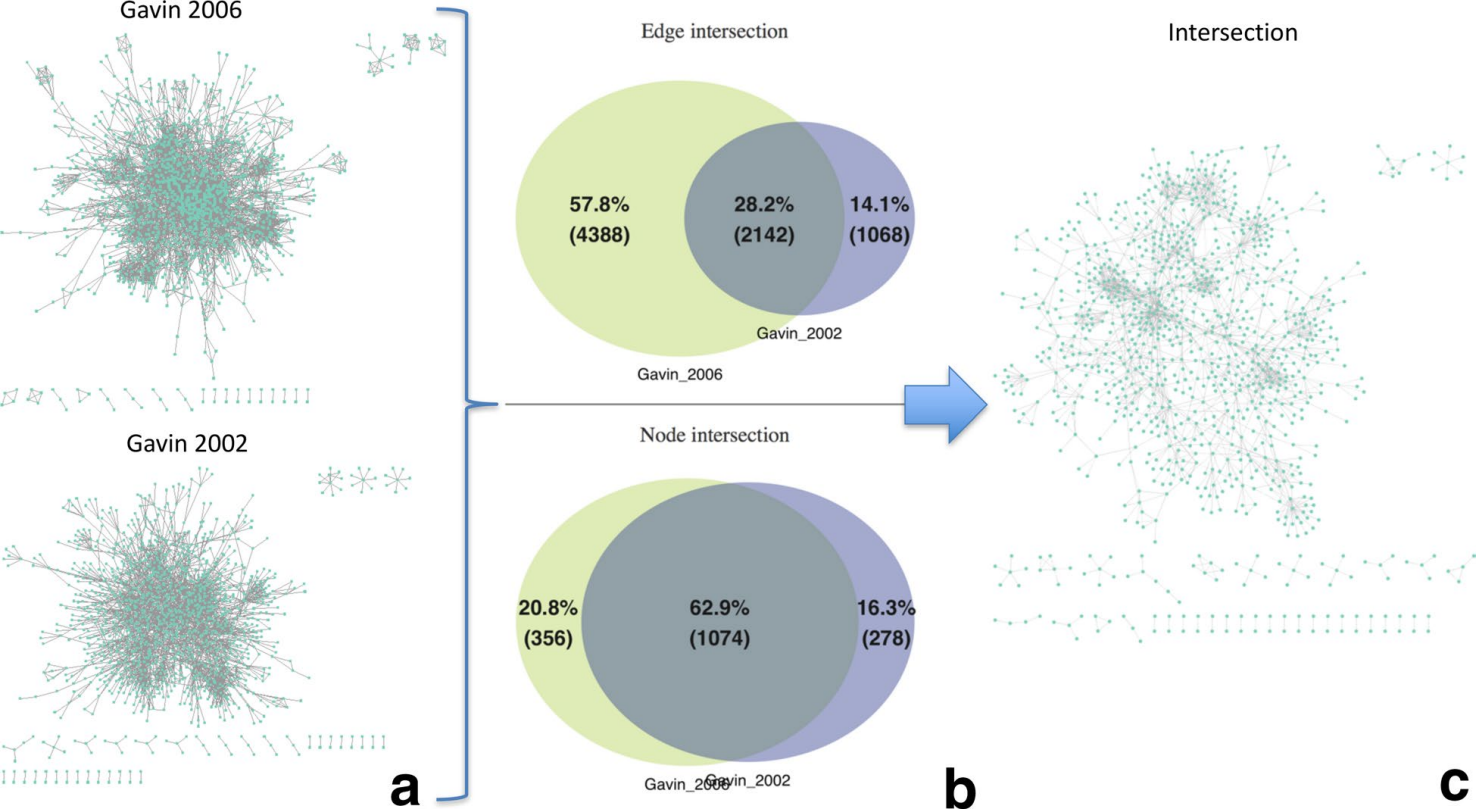
NAP’s functionality to find the intersection between ant pair of selected networks. **a** Gavin 2006 and 2002 PPI datasets visualized by Cytoscape 3.4.0 using the Prefuse layout. **b** NAP’s generated Venn diagrams showing the overlapping nodes and edges of the two networks. **c** NAP’s intersection export function and visualization with Cytoscape

Users can automatically find the intersection between any pair of selected networks. Once two networks have been selected, two Venn diagrams will be generated showing the node and the edge overlap between the two selected networks. In order to visualize the intersecting part of the networks, users can download the network in a CVS format and import it to third-party applications such as Cytoscape or Gephi. Figure 5 shows an example of how to find the intersection between Gavin 2002 and Gavin 2006 PPI datasets.

### Bipartite graphs

NAP is able to manage bipartite graphs. Given a bipartite graph, users can automatically extract its two monopartite projections and analyze them separately. In a gene–disease bipartite graph for example, one can generate a disease–disease network through common genes and vice versa, a gene–gene network through common diseases.

### Implementation

NAP’s web interface is written in Shiny and back-end functions implemented in R. Topological features are calculated with the use of igraph-R library [13] and plots are generated through R and plotly [21]. Static network visualizations are offered by the d3 library whereas dynamic network visualization is provided by CytoscapeWeb/Cytoscape.js [17, 18].

## Discussion

Network Analysis Provider (NAP) is designed to complement existing state-of-the-art visualization and analysis tools. It emphasizes on topological network analysis and inter-/intra-network topological feature comparison. Overall, we believe that NAP can reach users beyond the broader network analysis community and aid non-experts in analyzing their networks in a simplified and highly interactive way.

## Limitations

NAP runs on a browser and therefore, it is not optimized for large-scale networks. NAP’s future versions will include a much richer and optimized set of clustering algorithms [22], richer motif extraction algorithms, network alignment methods such as Corbi [23] and GraphAlignment [24], more scalable visualization, user account profiles to store and load the networks, incorporation of Arena3D [25, 26] for 3D multilayered network visualization and better handling of bipartite graphs taking into account their special topological properties.
